# Commissions in Lunacy

**Published:** 1851-01-01

**Authors:** 


### COMMISSIONS IN LUNACY.

A commission *de lunatico inquirendo* was held at the Sussex Arms Hotel, Hammersmith, before F. Barlow, esq., one of the masters in lunacy, relative to the state of Captain James Gordon, late of the 3rd West India Regiment, and now resident with, and under the care of, Dr. Forbes Winslow, of Sussex House, Hammersmith. The commission was issued on the petition of his wife, and was made imperative in consequence of the death of Sir Thomas McKenny, bart., the late father of Mrs. Gordon.

It appeared, from the statement of counsel, that Captain Gordon had been under Dr. Winslow's care since January, 1846. Prior to that period, he had been in two other establishments, one of which was that of Dr. Fox, of Bristol. He was subsequently placed in the private family of a physician, and becoming unmanageable, he was then removed to a cottage in St. John's Wood, and from thence he was taken to Sussex House.

*Dr. Forbes Winslow* was then examined at some length. Dr. Winslow represented that Captain Gordon laboured under several delusions. He believed that he was a man of title, and signs himself so; he thought that a conspiracy existed against him, and that the Roman Catholics were connected with it. He disowned his own wife, saying that he was not certain that she was his wife. In conjunction with these fancies, he is subject to violent paroxysms of mania, which come on without any assignable cause. At these times he is highly dangerous to himself and others. Dr. Winslow said that occasionally Captain Gordon was quite incoherent in conversation. He was incapable of exercising continuity of thought. It was extremely difficult to direct his attention to any one particular idea. He did not appear (although distinctly told) to have a clear notion of the nature of the inquiry. After the evidence of Dr. Winslow, Captain Gordon was introduced into the room, and took his seat by the side of the commissioner. Mr. Barlow then informed him of the nature of the investigation, and recapitulated the evidence of Dr. Winslow, with a view of exacting from Captain Gordon an explanation of the facts sworn to. It was, however, at once evident to the jury that the alleged lunatic could not advance anything to satisfy them as to his sanity. He admitted that he was violent, and this was owing to the air affecting his head. He was asked if he had anything to complain of. He replied, that he ought not to be deprived of his liberty, that he wished to go to the north; but would have nothing to do with his wife, whom he said was Lady Gordon.

Several members of the jury questioned Dr. Winslow as to his particular treatment of the case, with the view of ascertaining whether there were not causes, apart from his malady, which probably might originate the violence and excitement to which Captain Gordon was occasionally liable. Dr. Winslow said that Captain Gordon had, since the first day he was placed under his care, been humoured very much. He was invariably treated with the greatest kindness, and never contradicted; and although he now and then broke his windows, Dr. Winslow thought it unnecessary to have them barred or protected by wire gauze, considering that, in a temperament like Captain Gordon's, it would greatly increase his irritability and excitement. The jury expressed themselves to be perfectly satisfied with Dr. Winslow's explanation.

*Mr. Warwick*, a surgeon, and *Mr. Flint South*, vice-president of the College of Surgeons, and surgeon to St. Thomas's Hospital, were also examined, and gave evidence similar to Dr. Winslow's. They had no doubt of Captain Gordon's lunacy, and incapability of managing himself or affairs. Mr. Vickers, a relation of Captain Gordon, also gave similar evidence. A solicitor appeared, at the request of Captain Gordon, the supposed lunatic, to watch the proceedings. This gentleman appeared satisfied with the evidence adduced. The jury, without a minute's deliberation, declared Captain Gordon to be of unsound mind, and incapable of managing himself or affairs. The lunacy was carried back to the 3rd of January, 1846. It appears that Captain Gordon, independently of his half-pay, is entitled to two sums of 6000*l*. each, besides other money, and a legacy of 500*l*., left him by a sister, which is to be appropriated for his benefit.—(*Daily News*.)

---

Another *Commission of Lunacy* was held before the same master, in the neighbourhood of St. George's Circus, Blackfriars-road, relative to the state of mind of *Miss Teresa Wakeman*, residing in that locality, in the house of Mr. Solly, a chemist.

*Mr. Follett* appeared in support of the commission, which had been issued on the petition of the sister.

*Dr. Forbes Winslow* was first examined. It appears that he had received instructions to visit Miss Teresa Wakeman; but on arriving at the house, and sending up his card, she refused to see him. The matter was then brought before the Lord Chancellor, who gave Dr. Winslow full authority to obtain admission to the alleged lunatic. The following is the substance of Dr. Winslow's evidence:—

On the 28th February last, and on 4th of March inst., he visited the above *Mary Teresa Wakeman*, residing in the house of Mr. Solly, chemist, of No. 3, St. George's Circus, Blackfriars-road, in the county of Surrey, and that on both occasions he was accompanied by Mr. Laurie, a solicitor, who was present during the whole of the first interview with the said Mary Teresa Wakeman, and during a portion of the last examination of her on the 4th March.

That on said 28th day of February, said Mary Teresa Wakeman expressed herself in strong language against Mrs. Moorley, her sister, declaring that she was not her sister; and upon being asked whether she meant that she did not act in a sisterly way, or was not literally any relation of hers; she replied that she could not ascertain the fact until she had seen the parchments. She also said she had no brother, that the party said to be her brother was no relation of hers. She declared she was a special subject of persecution; and that the Roman Catholics were at the bottom of it. That the Roman Catholics had taken forcible possession of her parchments and papers, and until they were restored she could not reply satisfactorily to any questions. Upon being asked by witness whether she knew the amount of her money or her income, she replied she did not; to the question whether she had money in the funds, she said she did not know: and when Mr. Laurie told her that she had money, she said yes, but could give me no idea of the amount, whether it was 100*l*., or 1000*l*., or 10,000*l*. She had no idea of the amount of money standing in the funds in her name. Neither did she appear able to say what her income was, or what she was in the habit of receiving, or what she expected. She said that the Roman Catholics knew all about her money, for they were building churches and schools with it, particularly one in St. John's Wood.

That on the 4th March, when Dr. Winslow again saw the said Mary Teresa Wakeman, her conversation occasionally was wild and incoherent, and that she talked about her having directly descended from Edward the Second. She said that Edward the Second was awake, and thus the Wakemans sprung out of royalty. She said that Mrs. Moorley was engaged with the "Moon Works;" and upon being asked what she meant by the "Moon Works," she replied, if he had studied astrology witness would have known the "Moon Works" were managed by the Wakemans, and the Roman Catholics. She then referred to the Roman Catholics, whom she believes were persecuting her and have possession of her property; when asked again as to the amount of her income, she said the bondholders knew and would tell. She did not know whether her income is hundreds or thousands of pounds a year: she said that the Lord Chancellor has the control of it, and that he will inform witness as to the precise amount. She said she had never given offence to government, and she did not see why the government or Roman Catholics should interfere with her and her property. The Roman Catholics had concealed the Wakeman family papers, and she was in ignorance of her pecuniary resources, and of her family, until they were restored to her.

That she declared that the Duke of Wellington had been killed at Waterloo, and that her father was now the Duke of Wellington.

That upon being again questioned as to her sister and brother, whom he, Dr. Winslow, understood to be alive, she declared that she was an only child, and had no relatives or friends living.

That it appears that she is under the impression that she is a special subject of persecution by the Roman Catholics, who possess her private papers, and are building cathedrals and schools with her money; that she has no relations in the world; that her father is or was the Duke of Wellington; that what she calls the "Moon Works," and "Sun Works," are conspiring against her, and that the government and the Lord Chancellor know the amount of her pecuniary means, and control partly her property.

That her mind is evidently in a very weak and unsound state, and that witness considers her to be quite incapable of taking care of herself, or of managing her affairs.

*Mr. J. Bowling*, surgeon, of Hammersmith, was next examined.—His evidence was similar to that adduced by the previous witness.

*Miss Wakeman* was introduced to the jury, and in answer to several questions put to her by the commissioner, she gave very incoherent answers. The jury, without any hesitation, gave a verdict of unsoundness of mind.

---

